# Machine learning for the prediction of molecular dipole moments obtained by density functional theory

**DOI:** 10.1186/s13321-018-0296-5

**Published:** 2018-08-22

**Authors:** Florbela Pereira, João Aires-de-Sousa

**Affiliations:** LAQV and REQUIMTE, Departamento de Química, Faculdade de Ciências e Tecnologia, Universidade Nova de Lisboa, 2829-516 Caparica, Portugal

**Keywords:** Density functional theory (DFT), Molecular dipole moment, Quantitative structure property relationships (QSPR), Machine learning (ML), Partial atomic charges

## Abstract

Machine learning (ML) algorithms were explored for the fast estimation of molecular dipole moments calculated by density functional theory (DFT) by B3LYP/6-31G(d,p) on the basis of molecular descriptors generated from DFT-optimized geometries and partial atomic charges obtained by empirical or ML schemes. A database was used with 10,071 structures, new molecular descriptors were designed and the models were validated with external test sets. Several ML algorithms were screened. Random forest regression models predicted an external test set of 3368 compounds achieving mean absolute error up to 0.44 D. The results represent a significant improvement of the dipole moments calculated using empirical point charges located at the nucleus, even assuming the DFT-optimized geometry (root mean square error, RMSE, of 0.68 D vs. 1.53 D and R^2^ = 0.87 vs. 0.66).

**Electronic supplementary material:**

The online version of this article (10.1186/s13321-018-0296-5) contains supplementary material, which is available to authorized users.

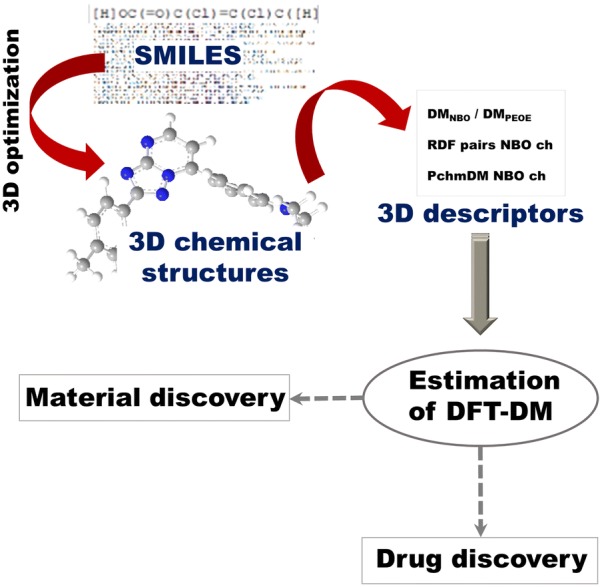

## Background

The dipole moment (DM) is a widely used parameter, which has been shown to explain observable chemical and physical properties of molecules in many different contexts. Its application in drug discovery, as well as the development of new materials currently attracts high interest [1, 10]. The DM has been useful in the assessment of cell permeability and oral bioavailability of drugs, as compounds with large dipole moments are generally more soluble in water and less likely to be absorbed through lipophilic membranes [1, 2]. In two benchmarking studies, one with a collection of 467 marketed orally available drugs [1], and the other with 1382 small drugs [2], it was observed that approximately 95% of drugs have DMs lower than 13 D and 10 D, respectively. The same parameter has been used to explain the catalytic activity of enzymes [3], and is commonly included as a descriptor in Quantitative Structure-Activity Relationships (QSAR) or Quantitative Structure-Property Relationships (QSPR) studies—and often found to be a highly relevant descriptor in the best models. A few recent examples are cited here [4–7]. In QSAR modeling of aromatase inhibition [4], antifungal activity [5], and HIV-1 protease/cyclin-dependent kinases inhibition [7], the molecular DM played a pivotal role as descriptor; it was calculated by molecular mechanics with the SYBYL program [4], or with DFT at the B3LYP/6-31G(d,p) [5] or B3LYP/6-31+G**(6d, 7f) [7] theory levels. An example of a QSPR model employing the DM descriptor is the estimation of micellar properties such as drug loading capacity (LC) [6], for which electronic structure factors and the DM were identified as the most important descriptors; in this case the DM was calculated with the DFT B3LYP functional and the 6-311G basis set.

A recent strategy for the design of mechanochromic luminogens was reported based on the DM of donor–acceptor molecules, using a series of 2,7-diaryl-[1,2,4]triazolo[1,5-a]pyrimidine derivatives; the DM was employed to explain and further predict the mechanochromic trends, which allowed the authors to design seven pairs of isomers with opposite mechanochromic trends [8]. Hyperpolarizability and dipole moments are crucial properties of organic non-linear optical materials [9]; although, in general, the hyperpolarizabilities depend on the difference between the excited and ground state DMs, in some cases they are proportional to the magnitude of ground sate dipole moments [10].

The application of quantum chemical calculations in organic materials discovery is well established, e.g. to predict electronic properties crucial in the design of organic photovoltaic materials (OPVs), organic-based flow batteries, and organic light-emitting diodes (OLED) [13–15]. Several projects related to drug discovery have also employed quantum chemistry calculations; examples include the prediction of protein-ligand interactions and binding energies [11], definition of drug-like chemical spaces [2], and building of large-scale databases of molecular structures and properties [12].

DMs calculated by DFT B3LYP functional hybrid approach revealed an excellent correlation (R^2^ = 0.952) [2] with experimental values in a data set of 200 small molecules from the CRC Handbook of Chemistry and Physics. Faber et al. [16] reported a mean absolute error of 0.10 D between B3LYP calculations and experiment using 49 molecules. Others have also reported good agreement between experimental and DFT calculated DMs for more complex molecules [17, 18]. Experimental measurements and quantum chemistry calculations were performed in the gas phase.

However, DFT calculations are still computationally too demanding for most large-scale virtual screening explorations, or for the incorporation in fast QSAR or QSPR models. Alternatives have been proposed including the derivation of DMs from available 3D molecular models and partial atomic charges obtained with empirical or ML methods. Rai and Bakken [19] reported fast and accurate models (random forests) to generate ab initio quality electrostatic potential (ESP) atomic charges, in which atomic descriptors were calculated from the 3D geometries of the molecules, and these charges were used to reproduce the quantum mechanical DMs (average absolute deviation of 1.2 D). Faber et al. [16] reported ML models to predict dipole moments (among other electronic ground-state properties) calculated by DFT at the B3LYP/6-31G(2df,p) level of theory. Screening of several regressor algorithms combined with various molecular representation schemes revealed a best model for DM, based on a graph convolution neural network and molecular graph representation, which achieved a MAE of 0.101 D for a test set of 13,000 molecules. The training set included *ca.* 118,000 organic molecules with up to nine heavy atoms limited to atomic elements H, C, O, N, and F [20]. In the same study, random forests yielded MAE between 0.434 D and 0.608 D.

ML from data precalculated by DFT or ab initio methods has emerged as a successful approach to deliver properties of atoms, bonds and molecules with high accuracy at a speed several orders of magnitude higher than would be obtained with the former methods [16, 21]. This requires computationally inexpensive molecular descriptors, available ML algorithms, and well-designed large data sets. ML models are expected to provide early stage filters that can identify promising molecules for further screening, e.g., by computationally more intensive methods.

Here we report the exploration of ML tools to predict dipole moments of organic molecules, using a database calculated by B3LYP/6-31G(d,p) for 10,071 structures with atomic elements H, C, N, O, F, S, Cl, Br, P, molecular descriptors based on the DFT-optimized geometries and available schemes for partial atomic charges. The predictions obtained by ML models are also compared with the DM values calculated assuming point charges located at the nucleus of atoms in the DFT geometry, and using partial atomic charges available from empirical and data-driven methods. The DM is a geometry-dependent property and the generation/optimization of the 3D structure is exterior to the ML models here described—these were trained to predict the DM for a specific given 3D geometry. DM predictions require a ML model and a 3D structure. If a 3D structure is unavailable, the accuracy of the predictions will depend on the ML model and on the quality of the 3D structure (obtained by some method independent of the ML model). The possibility of applying the models to external test sets with unknown 3D structures was also assessed by comparing DFT-calculated DM values with ML predictions obtained for 3D structures simulated by empirical methods. The “empirical methods” mentioned in this work to generate 3D structures do not use quantum chemistry calculations, but apply rules and molecular mechanics instead.

## Methods

### Data sets/selection of training and test sets

Molecular structures were retrieved from the ZINC database [22] and the GDB-13 database [23] of small organic molecules containing up to 7 atoms of C, N, O, F, S, Cl and Br. The structures were standardized with ChemAxon Standardizer (JChem 15.4.6, 2015, ChemAxon Ltd., Budapest, Hungary) and OpenBabel (Open Babel Package, version 2.3.1 http://openbabel.org) for neutralization and inclusion of all hydrogen atoms. Duplicated molecules were discarded, based on canonical SMILES and InChI codes (stereoisomers were considered as duplicated structures). The final database consisted of 10,071 molecules with molecular weights (MWs) in the range 40–251 g/mol, and containing up to 19 atoms of elements C, N, O, F, S, Cl, Br, and P. The database was randomly divided into a training set of 6703 molecules, and a test set of 3368 molecules. Two other external data sets (i.e. test set I and test set II) were also compiled from a benchmarking study [2] and an investigation of mechanochromic materials [8], which comprise 200 small organic molecules with MWs and number of heavy atoms in the range 27–250 g/mol and 2–10, respectively [calculated at the B3LYP/6-31G(d,p) level] and a series of 16 derivatives of 2,7-diaryl-[1,2,4]triazolo[1,5-a]pyrimidine [calculated at the B3LYP/6-31G(d) level] containing up to 32 heavy atoms and with a MWs in the range 272–451 g/mol, respectively.

### Geometry optimization and DFT calculations

The calculation of the molecular DM by DFT methods was performed in a semiautomatic way. Starting from SMILES strings or SDF files, the workflow consisted in the generation of the most stable conformer with JChem CXCALC (JChem 15.4.6, 2015, ChemAxon Ltd., Budapest, Hungary), optimization of the 3D structure with the GAMESS program [24, 25] using the hybrid B3LYP method [26, 27] and the 6-31G(d,p) basis set, followed by the calculation of the harmonic vibrational frequencies to confirm that the optimized geometry is a minimum on the potential energy surface (all real frequencies) at the same theory level. The molecular DM values were extracted directly from the GAMESS output. Their values range from 0.00 D to 13.18 D, with a mean of 2.91 D and MAD of 1.46 D. The optimized molecular structures and dipole moments were deposited in a public repository [28].

Three-dimensional models of the molecular structures from the test sets I and II were generated with empirical less computationally expensive tools such as CORINA version 2.4 (Molecular Networks GmbH, Erlangen, Germany) and Dreiding force field methods (JChem 15.4.6, 2015, ChemAxon, http://www.chemaxon.com).

### Calculation of molecular descriptors

The dipole moments defined by Eq. (1) were calculated from the DFT-optimized structures and two alternative partial atomic charges—natural bond orbital (NBO) partial atomic charges estimated using a ML tool developed in our lab (http://joao.airesdesousa.com/charges) [29], and Gasteiger partial equalization of orbital electronegativity (PEOE) partial atomic charges calculated by the ChemAxon CXCALC tool (JChem 15.4.6, 2015, ChemAxon Ltd., Budapest, Hungary):

1 $$\vec{\mu } = \mathop \sum \limits_{i = 1}^{n} q_{i} \vec{r}_{i}$$

where *μ* is the DM vector, *q*_*i*_ is the partial atomic charge of the atom *i*, and *r*_*i*_ is a vector from the center of mass to the charge q_i_. The two resulting values, DM_NBO_ and DM_PEOE_, were used as molecular descriptors. Only molecules belonging to the applicability domain of the models to estimate NBO charges [29] were included in the training and test sets. The training and test sets only included molecules belonging to the applicability domain of the models to estimate NBO charges, defined on the basis of the existence of all atom types in the training set of those models; the atom types were specified in terms of the element and number of H and non-H neighbors [29].

#### Radial distribution function (RDF) pair descriptors [30]

The 3D RDF descriptors were calculated by sampling the function of Eq. (2) at 128 equally distributed values of r between 0 and 12.8 Å:

2 $$RDF\left( r \right) = \mathop \sum \limits_{i = 1}^{N - 1} \mathop \sum \limits_{j = 1 + 1}^{N} p_{i} p_{j} e^{{ - B \left( {r - r_{ij} } \right)^{2} }}$$

where *N* is the number of atoms in the molecule, *p*_*i*_ is the charge of atom *i*, *B* is a fuzziness parameter (it was 100 in this study), and *r*_*ij*_ is the 3D distance between atoms *i* and *j*. Three sets of 128 RDF descriptors were separately calculated, derived from atom pairs with (a) a positive and a negative charge, (b) two positive charges, and (c) two negative charges. Both NBO and PEOE charges were used: RDF_N and RDF_P, respectively.

#### Projections charges/masses along DM axis (PchmDM)

These descriptors were designed to codify the distribution of the charges (and of the masses) projected along the axis of the dipole moment DM_NBO_ or DM_PEOE_. PchmDM descriptors were defined as the sum of all charges (or masses) projected onto each of 60 intervals of size 0.5 Å on the DM axis between − 15 Å and 15 Å with the origin assigned to the center of mass. Six series of 60 descriptors were generated (360 descriptors): (1) desc: sum of all charges at each interval; (2) desc_plus: desc descriptor restricted to positive charges; (3) desc_minus: desc descriptor restricted to negative charges; (4) desc_noH: desc descriptor restricted to non-hydrogen atoms; (5) desc_H: desc descriptor restricted to hydrogen atoms; (6) desc_mass: sum of all atomic masses at each interval. Both NBO and PEOE charges were used: PchmDM_N and PchmDM_P, respectively.

#### Geometric CDK descriptors

The geometric descriptors of the Chemistry Development Kit were calculated with the CDK Descriptor Calculator GUI version 1.4.6 (http://www.rguha.net/code/java/cdkdesc.html): 9 gravitational indices (characterizing the mass distribution of the molecule), 7 moments of inertia (calculates the main moments of inertia, ratios of the main moments and the radius of gyration), and 2 Petitjean shape indices (the topological and geometric shape indices, both measure the anisotropy in a molecule).

#### Fingerprints

Different types of fingerprints with different sizes were calculated by PaDEL-Descriptor version 2.21 (http://www.yapcwsoft.com/dd/padeldescriptor/) [31] and explored: 166 MACCS (MACCS keys), 881 PubChem fingerprints (ftp://ftp.ncbi.nlm.nih.gov/pubchem/specifications/pubchem_fingerprints.txt), and 1024 CDK (circular fingerprints).

### Selection of descriptors

In the quest for QSPR models with the minimum possible number of descriptors, feature selection was performed based on the importance of descriptors assessed by random forests (mean decrease in accuracy measure) and the CFS (Correlation-based Feature Subset Selection) algorithm [32] implemented in Weka 3.7.12 [33]. In CFS, the heuristic takes into account the usefulness of individual descriptors for predicting the property (DM) together with the level of intercorrelation among them. The experiments were conducted with the AttributeSelectedClassifier routine of Weka with the CfsSubsetEval option for attribute evaluator and the BestFirst, GreedyStepwise or PSOSearch option as the search method.

### ML methods

#### Random forests (RF) [34, 35]

A random forest is implemented as an ensemble of unpruned regression trees which are created using bootstrap samples of the training set. For each individual tree, the best split at each node is defined using a randomly selected subset of descriptors. Each individual tree is created using a different training and validation set. A RF yields a final prediction for an object as the average of the predictions of the individual regression trees. The predictions obtained for the objects left out of the training are compared to the target values, and deviations are averaged in the out-of-bag (OOB) error estimation. In the experiments presented here, RFs were used for the development of regression models to estimate DM. RFs were grown with the R program [36], version 3.2.3 using the RandomForest library [37]. The number of trees in the forest was set to 500, the number of descriptors available for each node was optimized and the other parameters were used with default values.

#### Support vector machines (SVMs) [38]

SVMs map multidimensional data into a hyperspace (a boundary or hyperplane) through a nonlinear transformation (kernel function) and a linear regression is then applied in this space. The boundary is defined with examples of the training set—the support vectors. In this study, SVM models were explored with the Weka31 (version 3.7.12) implementation of the LIBSVM software [39]. The epsilon-SVM-regression type was chosen, the kernel function was the radial basis function with the default gamma parameter, and the parameter C was optimized in the range of 10–10,000 through cross-validation with the training set.

#### Multilayer perceptron (MLP)

The Weka [33] MLPRegressor package (version 3.7.12) was used to implement feed-forward neural networks. It trains a multilayer network with one hidden layer using Weka’s Optimization class by minimizing the squared error plus a quadratic penalty with the BFGS method. The MLPRegressor options were set as default, except the number of hidden units and the ridge parameter that were optimized in cross-validation experiments with the training set.

#### Gaussian radial basis function (RBF)

A RBF is a feed-forward neural network (NN) with three layers of nodes, the middle (hidden) layer being made up of Gaussian or asymmetric kernels. Only the weights between the hidden layer and the output layer are modified during training. A RBF NN can accomplish a highly nonlinear mapping from the input space onto the output space. It was used in this work as specifically implemented by the RBFRegressor class in Weka [33] version 3.7.12, with the options set as default, except the number of basis functions and the number of sigma parameters that were optimized in cross-validation experiments with the training set.

## Results and discussion

In a first experiment, the ability of the NBO and PEOE partial atomic charges to directly calculate DMs was assessed, using Eq. (1) and the DFT-optimized geometry, and comparing the results with the DFT DMs—Table 1 and Additional file 1: Figure S1.

**Table 1:** Comparison of DFT DMs with DM_NBO_ and DM_PEOE_ for the 6703 and 3368 chemical structures of the training and test sets, respectively

Set	Charge^a^	MAE (D)	R^2^/RMSE (D)
Tr^b^	NBO	1.44	0.626/1.80
	PEOE	0.988	0.650/1.53
Te^c^	NBO	1.44	0.656/1.78
	PEOE	0.968	0.659/1.53

^a^The DMs were calculated using the DFT geometry optimization

^b^Training data set

^c^Test data set

There is a clear correlation between the empirical calculations and the DFT results, but we expected that these results could be significantly improved with the help of ML. It is noteworthy that although the deviations obtained with DM_NBO_ and DM_PEOE_ are similar, the inter-correlation between them is relatively low (R^2^ = 0.44) for the training set, which suggests that both parameters may be complementary and relevant as attributes in a ML approach to predict DFT DMs.

Of course, the application of Eq. (1), which considers charges of atoms located at their nucleus, can only be seen as an approximation to the DM calculated by DFT methods. Furthermore, two additional sources of deviations are the uncertainty regarding the 3D geometry and the quality of the charges. In the results of Table 1, the uncertainty from the geometry was avoided, as the DFT-optimized geometry was used, and the partial atomic charges are calculated independent of the geometry. Both PEOE and ML NBO charges were not developed to fit electrostatic potentials or dipole moments, but rather to account for intramolecular effects, e.g. related to reactivity or spectroscopic properties [29]. Therefore, we aimed at ML models that can build on the information contained in such charges, by learning from large datasets of DMs calculated with DFT calculations, to improve the ability to predict DMs.

A baseline performance for our models was estimated using the Zero Rule algorithm in a ten-fold cross-validation experiment with the training set in Weka, which yielded values for R, RMSE and MAE of − 0.038, 1.841 eV, and 1.447 eV, respectively.

### Random forest prediction of molecular DMs

Our ML strategy to predict DMs assumes that the molecular geometry is known from the onset, *i.e.*, the DFT-optimized geometry is used to calculate the 3D molecular descriptors that are presented to the regressors. So, the models are trained to predict the DM for that geometry. The results in Table 1 have shown that this is a task for which common methods are rather limited. Random forests regression models were trained with 6703 molecules represented by the descriptors RDF (with NBO and PEOE charges), PchmDM (with NBO and PEOE charges), geometric CDK descriptors, and other well-established fingerprints such as fragment fingerprints (166 MACCS keys and 881 PubChem) and circular fingerprints (CDK). The models were validated with the independent test set consisting of 3368 molecules—Table 2. Although the predictive power of the eight models developed (Table 2) was very modest, with RMSE in the range of 1.2–1.4 D for the test set, the best sets of descriptors and fingerprints were selected for further experiments. After the exploration of models derived with 3D molecular descriptors and fingerprints, we investigated the inclusion of the descriptors DM_NBO_ and DM_PEOE_ to the best sets of descriptors and fingerprints—Table 3.

**Table 2:** Prediction of the DFT DM by random forests on the basis of different molecular descriptors

Descriptors (#)	Training set^a^		Test set	
	MAE (D)	R^2^/RMSE (D)	MAE (D)	R^2^/RMSE (D)
RDF_N^b^ (384)	0.944	0.480/1.332	0.947	0.498/1.344
RDF_P^c^ (384)	0.890	0.512/1.295	0.882	0.549/1.287
PchmDM_N^b^ (360)	0.924	0.545/1.267	0.880	0.589/1.250
PchmDM_P^c^ (360)	0.873	0.569/1.240	0.931	0.566/1.278
CDK^d^ (47)	0.983	0.434/1.385	0.985	0.445/1.402
MACCS FP^e^ (166)	0.790	0.579/1.195	0.775	0.609/1.182
PubChem FP^e^ (881)	0.817	0.547/1.238	0.801	0.584/1.217
CDK FP^e^ (1024)	0.880	0.501/1.301	0.874	0.521/1.305

^a^OOB estimation

^b^Descriptors calculated using NBO charges

^c^Descriptors calculated using PEOE charges

^d^Geometric CDK descriptors

^e^Fingerprints

**Table 3:** Prediction of the DFT DM by random forests using DM_NBO_ or DM_PEOE_

Descriptors	Training set^a^		Test set	
	MAE (D)	R^2^/RMSE (D)	MAE (D)	R^2^/RMSE (D)
RDF_N + DM_NBO_^b^	0.639	0.747/0.930	0.638	0.761/0.929
RDF_P + DM_PEOE_^c^	0.624	0.740/0.946	0.615	0.765/0.929
PchmDM_N + DM_NBO_^b^	0.647	0.753/0.924	0.651	0.769/0.921
PchmDM_P + DM_PEOE_^c^	0.639	0.735/0.953	0.630	0.759/0.936
CDK + DM_NBO_^d^	0.713	0.705/1.00	0.700	0.724/0.990
CDK + DM_PEOE_^e^	0.708	0.685/1.03	0.704	0.705/1.02
MACCS + DM_NBO_	0.526	0.806/0.813	0.507	0.826/0.792
MACCS + DM_PEOE_	0.563	0.777/0.873	0.543	0.801/0.847

^a^OOB estimation

^b^Descriptors calculated using NBO charges, and DM_NBO_

^c^Descriptors calculated using PEOE charges, and DM_PEOE_

^d^Geometric CDK, and DM_NBO_

^e^Geometric CDK, and DM_PEOE_

The inclusion of both DM_NBO_ and DM_PEOE_ is advantageous, and the best performance was observed with the MACCS fingerprints combined with DM_NBO_. The good performance of 2D descriptors (and the observation that the most important 2D descriptors encode for small polar functional groups—see below) indicate that the presence of small polar fragments have a strong impact in the dipole moment of the whole molecule. Descriptors calculated with NBO charges performed slightly better than those with PEOE and were preferentially used in the following experiments. Combinations of 3D descriptors (RDF, PchmDM, CDK), 2D descriptors (MACCS fingerprints), DM_NBO_ and DM_PEOE_ were explored—Tables 4 and 5. The results reveal that the inclusion of CDK descriptors made almost no difference in the results, but increased the calculation time—they were therefore not included in the following experiments.

**Table 4:** Prediction of the DFT DM by random forests using NBO charges and combining different descriptors

Models	Training set^a^		Test set	
	MAE (D)	R^2^/RMSE (D)	MAE(D)	R^2^/RMSE (D)
A^b^	0.627	0.757/0.912	0.623	0.774/0.905
B^c^	0.616	0.762/0.903	0.611	0.778/0.896
C^d^	0.525	0.823/0.780	0.512	0.846/0.752
D^e^	0.522	0.824/0.777	0.509	0.846/0.750
E^f^	0.562	0.790/0.850	0.553	0.807/0.838
F^g^	0.497	0.837/0.749	0.479	0.860/0.719

^a^OOB estimation

^b^RDF pairs NBO charges, PchmDM NBO charges, and DM_NBO_

^c^RDF pairs NBO charges, PchmDM NBO charges, geometric CDK, and DM_NBO_

^d^RDF pairs NBO charges, PchmDM NBO charges, DM_PEOE_, and DM_NBO_

^e^RDF pairs NBO charges, PchmDM NBO charges, geometric CDK, DM_PEOE_, and DM_NBO_

^f^RDF pairs NBO charges, PchmDM NBO charges, MACCS fingerprints, and DM_NBO_

^g^RDF pairs NBO charges, PchmDM NBO charges, MACCS fingerprints, DM_PEOE_, and DM_NBO_

**Table 5:** Prediction of the DFT DM by random forests using a combination of DM_NBO_ and DM_PEOE_ with different type of descriptors

Models	Training set^a^		Test set	
	MAE (D)	R^2^/RMSE (D)	MAE(D)	R^2^/RMSE (D)
MACCS^b^	0.460	0.853/0.707	0.444	0.872/0.680
RDF^b,c^	0.533	0.817/0.791	0.522	0.839/0.767
PchmDM^b,d^	0.540	0.819/0.786	0.533	0.839/0.765
CDK^b^	0.538	0.815/0.793	0.525	0.837/0.765

^a^OOB estimation

^b^Combining DM_PEOE_, and DM_NBO_

^c^RDF pairs NBO charges

^d^PchmDM NBO charges

The best models (C and F) yielded MAE of 0.512 D and 0.479 D for the test set, which are approximately 35% and 33% of the MAD from the average (1.47 D). The models are also significantly more accurate than the baseline model described above.

In order to evaluate the robustness of the approach, model C was evaluated using four alternative random splits of the data set into training and test sets: the test set predictions exhibited R^2^, RMSE and MAE values in the intervals 0.82–0.85, 0.75–0.79 D and 0.51–0.53 D, respectively.

The combination of DM_NBO_ and DM_PEOE_ with different type of descriptors achieved the models with the best performance, as can be seen in Tables 4 and 5. However we wanted also to investigate how they perform with each type of descriptors e.g. MACCS FP, RDF (with NBO charges), PchmDM (with NBO charges), geometric CDK descriptors—Table 6.

**Table 6:** RF prediction of DM with subsets of descriptors from models C and F

Model/no descriptors	MAE (D)	R^2^/RMSE (D)
Training set		
C/75^a^	0.515	0.826/0.769
C/100^a^	0.517	0.826/0.771
C/125^a^	0.518	0.826/0.7709
C/32^b^	0.521	0.824/0.773
C/39^c^	0.523	0.824/0.775
C/296^d^	0.529	0.821/0.782
F/75^a^	0.482	0.844/0.731
F/100^a^	0.482	0.844/0.731
F/125^a^	0.483	0.844/0.732
F/34^b^	0.501	0.838/0.745
F/41^c^	0.499	0.838/0.743
F/297^d^	0.503	0.832/0.755
Test set		
C/75^a^	0.502	0.847/0.744
C/100^a^	0.506	0.846/0.748
C/125^a^	0.505	0.845/0.748
C/32^b^	0.509	0.845/0.747
C/39^c^	0.512	0.844/0.749
C/296^d^	0.519	0.843/0.758
F/75^a^	0.468	0.864/0.704
F/100^a^	0.466	0.865/0.702
F/125^a^	0.466	0.867/0.699
F/34^b^	0.481	0.860/0.713
F/41^c^	0.479	0.860/0.710
F/297^d^	0.485	0.852/0.725

^a^OOB estimation for the training set

^b^Using the mean decrease in accuracy measure of importance for the descriptors in the RF algorithm

^c^Using the the CFS with BestFirst routine from Weka

^d^Using the the CFS with GreedyStepwise routine from Weka

^e^Using the the CFS with PSOSearch routine from Weka

Simultaneous inclusion of the two DMs (DM_NBO_ and DM_PEOE_) to the different types of descriptors studied allowed to obtain models with a much greater predictive capacity than the one obtained without the inclusion of these DMs, for all the experiments the MAE that was obtained is less than or equal to 0.533 D. The best model, standing out in all the prediction parameters of the other models for training and test sets, was achieved using 2D descriptors, MACCS fingerprints. It was impressive for us the performance achieved by this model, yielded MAE of 0.444 D for the test set, using only the MACCS fingerprints and the two DMs calculated (DM_NBO_ and DM_PEOE_).

### Descriptor selection and optimization of QSPR methods

**Fig. 1:**
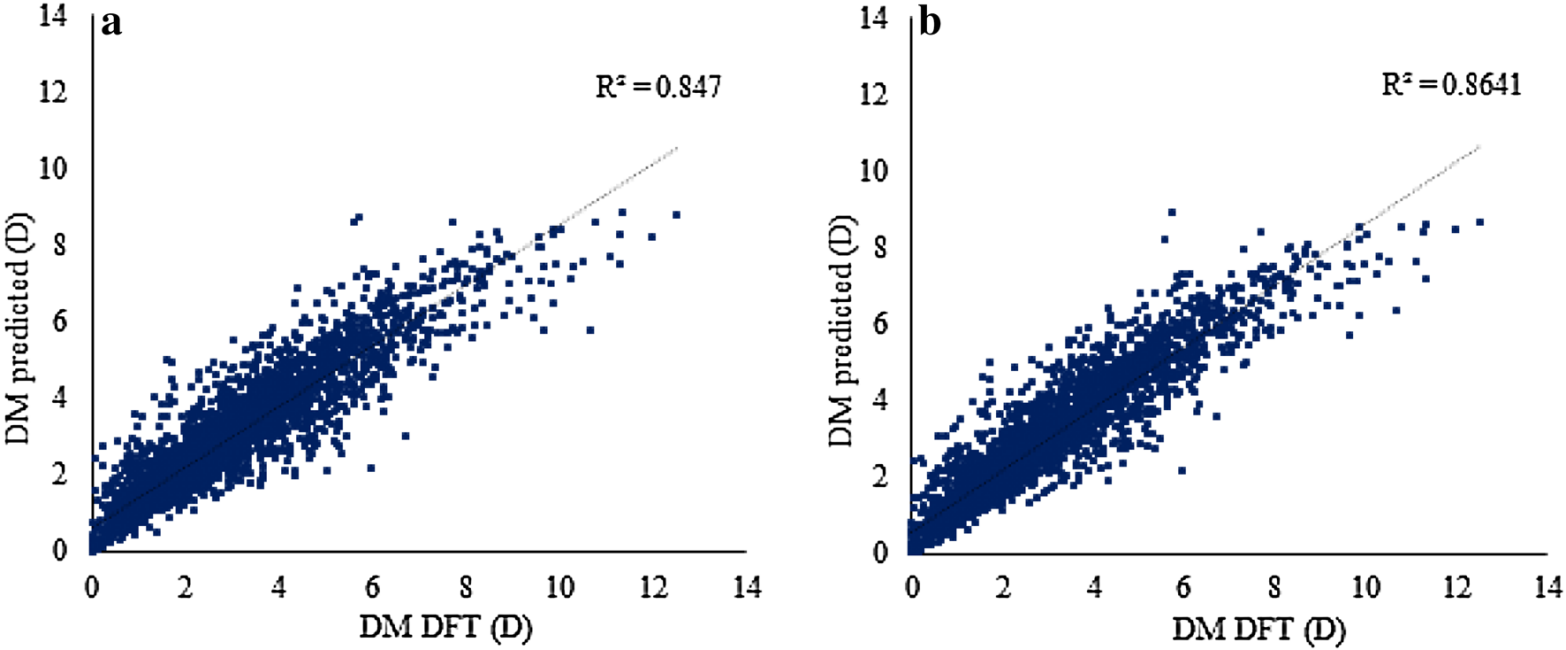
Predicted versus DFT-calculated DM for the 3368 molecular structures of the external test set. **a** Predictions obtained with the RF C model trained with 75 descriptors; **b** predictions obtained with the RF F model trained with 75 descriptors

Procedures for feature selection were applied to the descriptors of models C and F—Table 6. The 75 most important descriptors of models C and F were identified by RFs and enabled the training of new RF models with even better prediction accuracies than the models trained with the whole set of descriptors (746 and 912 descriptors, respectively). A graphical representation of the predictions versus the DFT-calculated values for approach C and F are shown in Fig. 1. The selected 75 descriptors for approach C included DM_NBO_ and DM_PEOE_, 58 RDF descriptors (26 of type a, 15 of type b, and 17 of type c), and 15 PchmDM descriptors (1 of type desc, 1 of type desc_plus, 1 of type desc_minus, 3 of type desc_noH, 3 of type desc_H, and 5 of type desc_mass). Within the top ten descriptors, DM_NBO_ is the most important descriptor, followed by DM_PEOE_ and RDFs of the three types (six of type a; one of type b in the 8th position; one of type c in the 5th position). The Pearson correlation coefficients between each of these eight RDF descriptors and the DFT DM are in the range of 0.10–0.45 for the training set.

Similarly, the inspection of the ten most important descriptors of model F revealed DM_NBO_ and DM_PEOE_ as the first and second most important descriptors, respectively. MACCS fingerprints occupy the 3rd, 5th and 10th positions, encoding the presence of nitrile, S = A groups (where A is any atom) and the N atom, respectively. The remaining positions are occupied by RDF descriptors of type a. The Pearson correlation coefficients between these MACCS descriptors and the DFT DM is in the range of 0.24-0.28 for the training set.

In an additional experiment, the RDF and PchmDM descriptors were discarded from model F, and a new model was re-trained only using MACCS, DM_NBO_ and DM_PEOE_ descriptors. The results were slightly improved (training set: MAE = 0.46 D, RMSE = 0.71, R^2^ = 0.85; test set: MAE = 0.44 D, RMSE = 0.68 D, R^2^ = 0.87) indicating that the two DM_NBO_ and DM_PEOE_ descriptors together incorporate the crucial 3D information to deliver the predictive ability of DFT DM here described (Additional file 1: Figure S2).

Further validation was performed using the *y*-randomization technique. The best C model was rebuilt with 5 modified training sets; the *y*-column data (DFT DM) was scrambled, keeping the descriptor matrix unchanged. The random models were found to have a considerably lower R^2^ and higher RMSE compared to those of the original model (R^2^ of 0.0005–0.0015 and RMSE of 1.89–1.91 D for the test set), further corroborating the statistical reliability of the model.

These results compare favorably to those reported by Lilienfeld and co-workers using a kernel ridge regression algorithm trained with 16,000 molecules to predict the DM calculated with the B3LYP/6-31g(2df,p) level of theory, which achieved a MAE of 0.63 D for an external test set [40]. More recently, in the some lab, a ML model developed with a graph convolution neural network and molecular graph representation, obtained a MAE of 0.101 D for a test set of 13,000 molecules [16]. In spite of the large number of molecules that comprise the data set (131,000 molecules), they are limited to five atomic elements (H, C, O, N, and F) and molecules containing up to 9 heavy atoms. The MAD of the DM is 1.17 D for the whole data set [20], which is smaller than for our data set (1.46 D). In order to evaluate the performance of our best C model with molecules made of the same elements, we retrained and re-validated the model without the molecules containing S, Br, Cl, or P. The MAD of the data set was reduced to 1.33 D, as was the MAE for the test set of 2667 molecules (0.42 D). The Rai and Bakken ML model to predict B3LYP/6-31G* electrostatic potential partial charges was applied to the calculation of dipole moments using Eq. (1), reporting a MAE of 1.2 D for an external test set of 5000 organic molecules [19]. However, this result is not directly comparable with ours since the DMs were processed as vectors and the deviation was calculated as the size of the difference vector.

### Exploration of other ML techniques

A comparison of three other state-of-the-art ML regressors—support vector machines (SVM), multilayer perceptron (MLP), and Gaussian Radial Basis Function (RBF)—is shown in Table 7, based on models built with the 75 most important descriptors previously identified for model C. Variation of the ML algorithm could not achieve any consistent improvement of the results obtained with random forests for the training and test sets.

**Table 7:** Exploration of different ML algorithms in the prediction of DFT DM using the 75 most important descriptors obtained for model C

ML	MAE (D)	R^2^/RMSE (D)
Training set		
SVM	0.531	0.819/0.783
MLP	0.550	0.813/0.797
RBF	0.561	0.811/0.801
Test set		
SVM	0.526	0.840/0.755
MLP	0.531	0.836/0.763
RBF	0.538	0.839/0.757

Predictions for the training set were obtained with ten-fold cross-validation experiments

### Application of the RF model without DFT-optimized geometries

**Fig. 2:**
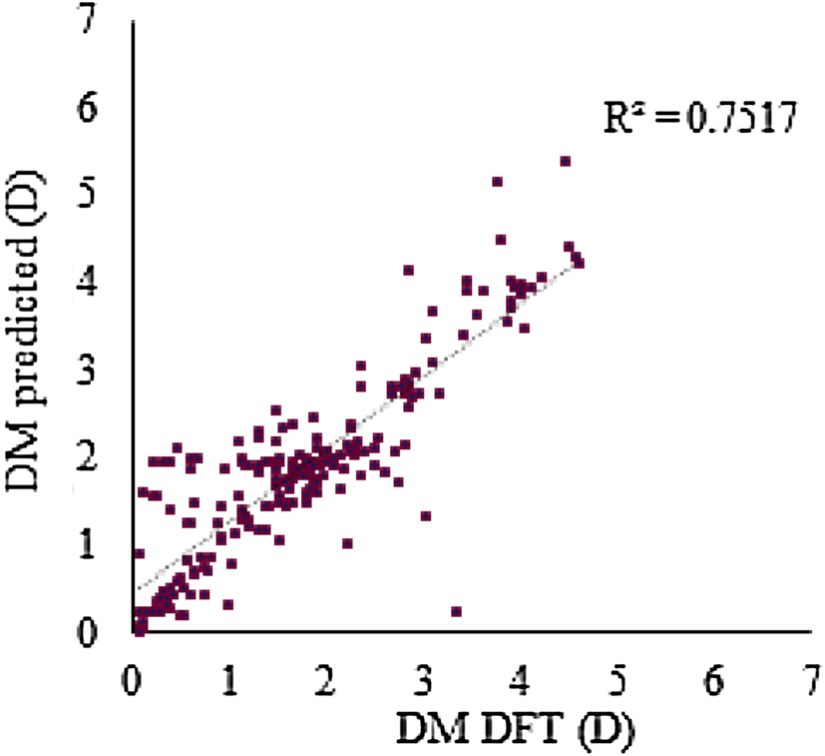
Predicted by the model MACCS_DM_NBO__DM_PEOE_ versus DFT-calculated DM [2] for the 200 molecular structures of the test set I

**Fig. 3:**
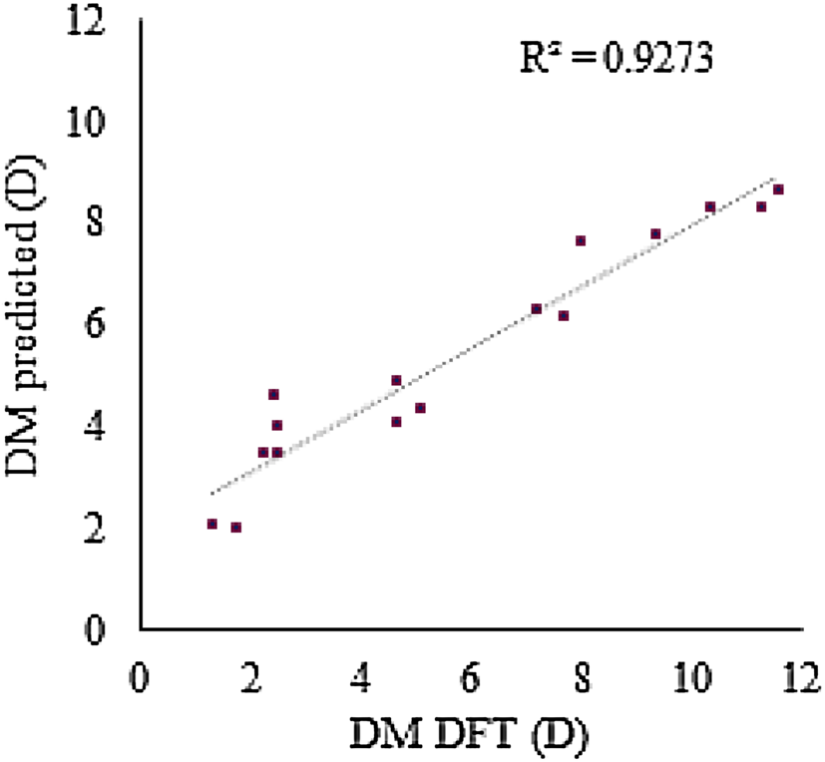
Predicted by the model MACCS_DM_NBO__DM_PEOE_ versus DFT-calculated DM [8] for the 16 molecular structures of the test set II

The predictive ability of the RF models for a data set in which the DFT-optimized structure is not available was evaluated. The 3D models were generated by CORINA or Dreiding force field methods for the test set, the molecular descriptors were calculated from these 3D structures, and the previously trained RF models were used to get predictions. The best RF models C, F and MACCS_DM_NBO__DM_PEOE_ had been trained with DFT-optimized geometries. The MAE increased to 0.78 D, 0.74 D and 0.68 D, respectively, and were essentially independent of the conformer generation method. The same methodology was followed with two new external data sets (i.e. test set I and test set II), consisting of 200 small organic molecules and a series of 16 2,7-diaryl-[1, 2, 4]triazolo[1,5-a]pyrimidine derivatives, respectively. The predictions were compared to the DM values calculated at the B3LYP/6-31G(d,p) and B3LYP/6-31G(d) levels, respectively, which were retrieved from the literature [2, 8]. The results are presented in Table 8, Figs. 2 and 3.

**Table 8:** Random forest prediction of the DFT DM for the external test sets I and II

Models/test sets	MAE (D)	R^2^/RMSE (D)
C/I^a^	0.559	0.598/0.782
F/I^a^	0.462	0.690/0.656
MACCS_DM_NBO__DM_PEOE_/I^a^	0.370	0.752/0.573
C/II^b^	1.362	0.938/1.63
F/II^b^	1.344	0.944/1.600
MACCS_DM_NBO__DM_PEOE_/II^b^	1.292	0.927/1.545

^a^Test set I comprising 200 molecules calculated at the B3LYP/6-31G(d,p) level

^b^Test set II comprising 16 molecules calculated at the B3LYP/6-31G(d) level

**Fig. 4:**
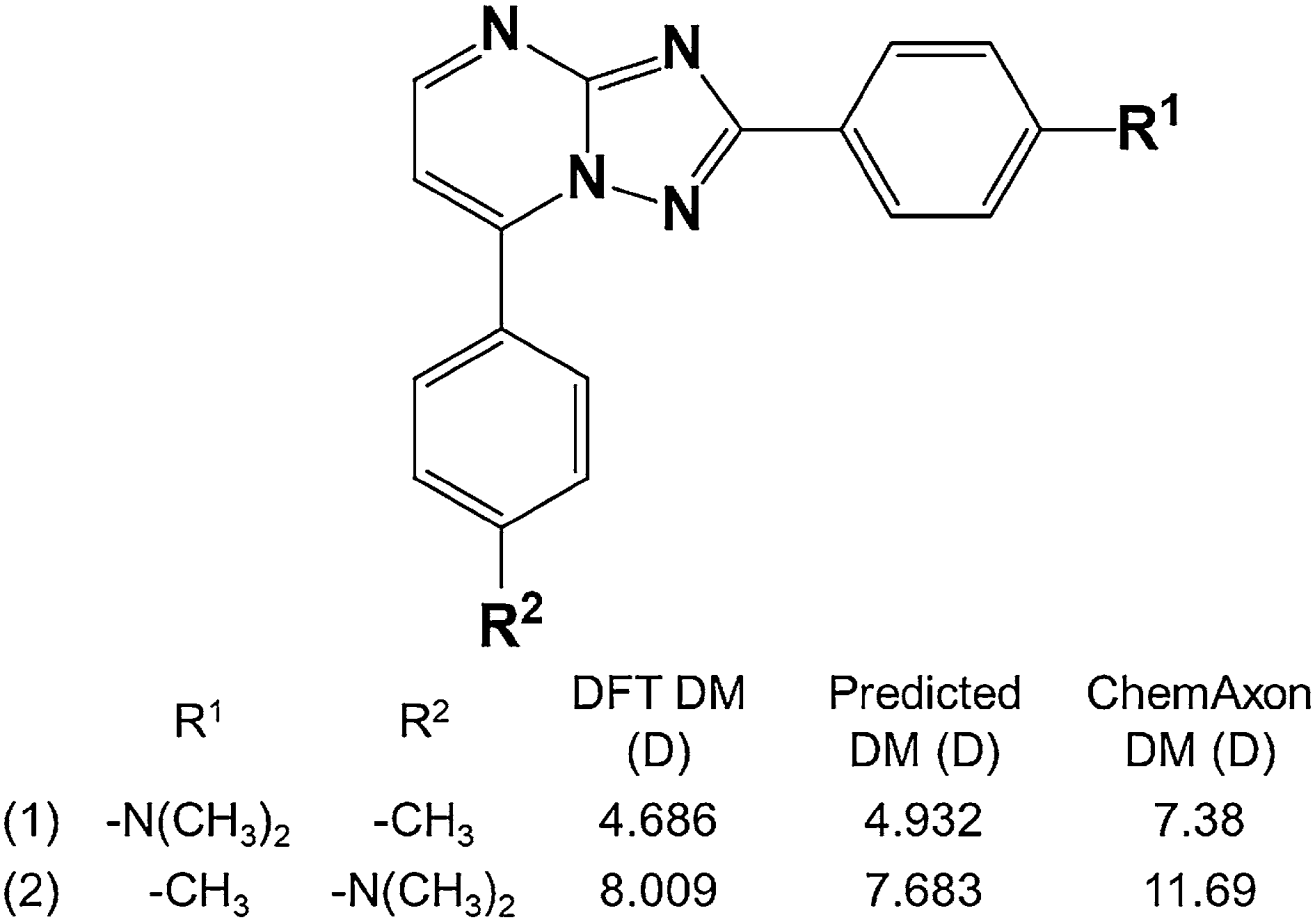
Illustration of predicted DM values for two molecules of test set II, obtained by the best RF MACCS_DM_NBO__DM_PEOE_ model, DFT calculations [8], and the ChemAxon CXCALC tool

A MAE of 0.37 D was achieved for test set I with model MACCS_DM_NBO__DM_PEOE_. Although the MAE is higher for test set II (due to a systematic deviation), the ability to predict trends is remarkable (R^2^ = 0.93), which is illustrated with the results for a case of DM cliffs in Fig. 4.

## Conclusions

ML models trained with 6703 molecules and the respective DMs calculated by density functional theory with B3LYP/31G(d,p) were able to reproduce DFT calculations with MAE up to 0.44 D for an external test set, which is *ca.* 30% of the MAD in the database. Random forests yielded the best results, and provided a subset of descriptors with high predictive power, notably MACCS fingerprints and the two empirical DMs based on Eq. (1) with point charges calculated by two different methods. The ML approach provides estimations of DMs significantly closer to DFT calculations than available empirical methods implementing Eq. (1). The ability to generate 3D structures is outside the scope of this paper; the models here reported have application in the prediction of the DM for a given 3D structure. As this article demonstrates, the available 3D structure is only one of the requirements to predict the DM. The application of the ML models (trained with DFT-optimized 3D structures) to molecules with 3D structures generated by empirical methods yielded predictions of the DFT dipoles with worse accuracies for external test sets, but even though, a high correlation was observed (R^2^ = 0.93) between predicted and calculated DFT DM for a series of 16 mechanochromic molecules.

## Additional file

**Additional file 1:** **Figure S1.** Graphical representation of the DFT-DM vs. a) DM_NBO_ and b) DM_PEOE_ for the test set. **Figure S2.** Predicted vs. DFT-calculated DM for the 3368 molecular structures of the test set using the model MACCS_DM_NBO__DM_PEOE_.
