# Supplementary material for: Machine learning for the prediction of molecular dipole moments obtained by density functional theory
Source: J Cheminform. 2018 Aug 22;10:43. doi: 10.1186/s13321-018-0296-5 (PMC6104469; doi:10.1186/s13321-018-0296-5)
Supplement: Supplementary file 1 — Additional file 1: Figure S1. Graphical representation of the DFT-DM vs. a) DMNBO and b) DMPEOE for the test set. Figure S2. Predicted vs. DFT-calculated DM for the 3368 molecular structures of the test set using the model MACCS_DMNBO_DMPEOE. [file 13321_2018_296_MOESM1_ESM.docx]

**SUPPORTING INFORMATION**

**Machine Learning for the Prediction of Molecular Dipole Moments Obtained by Density Functional Theory**

Florbela Pereira1 and João Aires-de-Sousa1*

^1^LAQV and REQUIMTE, Departamento de Química, Faculdade de Ciências e Tecnologia, Universidade Nova de Lisboa, 2829-516, Caparica, Portugal

^*^ Correspondence: [joao@airesdesousa.com](mailto:joao@airesdesousa.com)

Figure S1. Graphical representation of the DFT-DM vs. a) DM_NBO_ and b) DM_PEOE_ for the test set.

Figure S2. Predicted *vs*. DFT-calculated DM for the 3368 molecular structures of the test set using the model MACCS_ DM_NBO__DM_PEOE_.


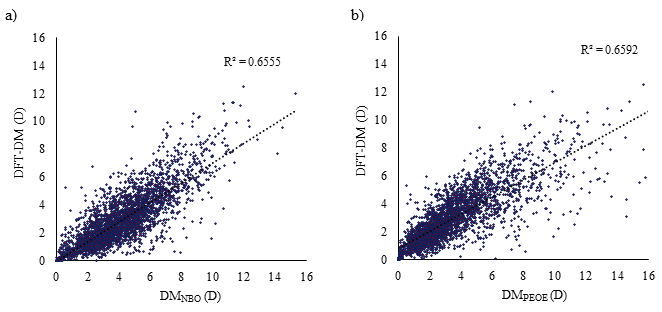


Figure S1. Graphical representation of the DFT-DM vs a) DM_NBO_ and b) DM_PEOE_ for the test set.


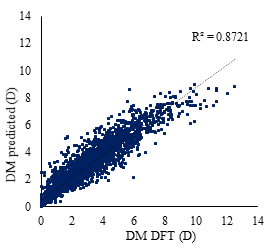


Figure S2. Predicted *vs*. DFT-calculated DM for the 3368 molecular structures of the test set using the model MACCS_ DM_NBO__DM_PEOE_.
